# Supplementary material for: Three-dimensional label-free imaging and analysis of Pinus pollen grains using optical diffraction tomography
Source: Sci Rep. 2018 Jan 29;8:1782. doi: 10.1038/s41598-018-20113-w (PMC5788986; doi:10.1038/s41598-018-20113-w)
Supplement: Supplementary file 1 — Supplementary Information [file 41598_2018_20113_MOESM1_ESM.pdf]

Supplementary Information

Three-dimensional label-free imaging and analysis of *Pinus* pollen grains using optical diffraction tomography

Geon Kim[a,b], SangYun Lee[a,b], Seungwoo Shin[a,b] and YongKeun Park[a,b,c,*]

[a]Korea Advanced Institute of Science and Technology (KAIST), Department of Physics, 291 Daehak-ro, 34141 Daejeon, Republic of Korea

[b]KAIST, KI for Health Science and Technology, 291 Daehak-ro, 34141 Daejeon, Republic of Korea

[c]Tomocube, Inc., 48 Yuseong-daero 1184beon-gil, 34109 Daejoen, Republic of Korea

***yk.park@kaist.ac.kr**

The following Supporting Information is available for this article:

Video S1. Three-dimensional rendering of a whole pollen grain from a Korean red pine plant. Rendering was generated using a commercial software (Tomostudio, Tomocube Inc., Republic of Korea) Refractive index (RI) between 1.5 and 1.515 is rendered in violet and RI between 1.525 and 1.54 is rendered in yellow-green. Cube length = 60 μm.

Video S2. Three-dimensional rendering of an exine shell from a Korean red pine plant. Refractive index between 1.5 and 1.515 is rendered in violet and RI between 1.525 and 1.54 is rendered in yellow-green. Cube length = 60 μm.

Video S3. Three-dimensional rendering of a whole pollen grain from a golden Korean red pine plant. Refractive index between 1.5 and 1.515 is depicted in rendered and RI between 1.525 and 1.54 is rendered in yellow-green. Cube length = 60 μm.

Video S4. Three-dimensional rendering of an exine shell from a golden Korean red pine plant. Refractive index between 1.5 and 1.515 is rendered in violet and RI between 1.525 and 1.54 is rendered in yellow-green. Cube length = 60 μm.

Video S5. Three-dimensional rendering of a whole pollen grain from a Japanese red pine plant. Refractive index between 1.5 and 1.515 is rendered in violet and RI between 1.525 and 1.54 is rendered in yellow-green. Cube length = 60 μm.

Video S6. Three-dimensional rendering of an exine shell from a Japanese red pine plant. Refractive index between 1.5 and 1.515 is rendered in violet and RI between 1.525 and 1.54 is

rendered in yellow-green. Cube length = 60 μm.

Video S1. Three-dimensional rendering of a whole pollen grain from a Japanese black pine plant. Refractive index between 1.5 and 1.515 is rendered in violet and RI between 1.525 and 1.54 is rendered in yellow-green. Cube length = 60 μm.

Video S2. Three-dimensional rendering of an exine shell from a Japanese black pine plant. Refractive index between 1.5 and 1.515 is rendered in violet and RI between 1.525 and 1.54 is rendered in yellow-green. Cube length = 60 μm.
